# Path Planning for USVs in Complex Marine Environments Based on an Improved Hybrid TD3 Algorithm

**DOI:** 10.3390/s26061823

**Published:** 2026-03-13

**Authors:** Zhenxing Zhang, Xiaohui Wang, Qiujie Wang, Mingwei Zhu, Mingkun Feng

**Affiliations:** 1 School of Computer Science and Technology, Zhejiang University of Science and Technology, Hangzhou 310023, China; 222408855057@zust.edu.cn; 2 School of Artificial Intelligence and Information Engineering, Zhejiang University of Science and Technology, Hangzhou 310023, China; 222408855031@zust.edu.cn (X.W.); 222308855049@zust.edu.cn (Q.W.); mkfeng96@zust.edu.cn (M.F.)

**Keywords:** path planning, ocean current disturbance, TD3, APF, CDA-PER, TPN

## Abstract

Real-time path planning for Unmanned Surface Vehicles (USVs) in complex marine environments remains challenging due to unstructured environments, ocean current disturbances, and dynamic obstacles. This paper proposes an improved Hybrid Safety and Reward-Sensitive Twin Delayed Deep Deterministic Policy Gradient (H_RS_TD3) algorithm and constructs a high-fidelity simulation environment based on GEBCO bathymetric data and CMEMS ocean current data. The path planning problem is formulated as a Markov Decision Process (MDP), where the state space incorporates multi-beam radar perception, ocean current disturbances, and relative goal information, while the action space outputs continuous thrust and rudder commands subject to vehicle dynamics constraints. The proposed framework integrates a risk-aware hybrid safety decision architecture, a Trajectory Predictor Network (TPN), a Curvature-driven Advantage-based Prioritized Experience Replay (CDA-PER) mechanism, and an uncertainty-aware conservative Q-learning strategy to enhance navigation safety, sample efficiency, and policy stability. Comprehensive simulations demonstrate that, compared with baseline deep reinforcement learning methods, the proposed approach achieves faster convergence, improved stability, and competitive path efficiency while consistently maintaining sufficient obstacle clearance and millisecond-level inference latency, validating its effectiveness and practical feasibility for safe USV navigation in realistic dynamic marine environments.

## 1. Introduction

With the increasing complexity of marine resource exploitation, environmental monitoring, maritime surveillance, patrol, and search-and-rescue missions. USV has attracted widespread attention due to its low cost, high efficiency, and strong environmental adaptability, particularly in autonomous marine applications [1]. USV has been extensively deployed in both civilian and military applications to replace human operators in hazardous missions [2]. Within autonomous navigation systems, efficient path planning algorithms constitute one of the core enabling technologies, aiming to generate safe, smooth, and energy-efficient collision-free trajectories for USVs operating in complex marine environments containing static obstacles, dynamic threats, and environmental disturbances [3].

Existing path planning approaches can generally be categorized into traditional methods and reinforcement learning-based methods. Classical algorithms such as A*, Dijkstra, and RRT [4,5,6] perform well in known static maps or relatively simple marine environments. However, when confronted with time-varying dynamic obstacles, these methods often suffer from high computational complexity, making it difficult to satisfy real-time requirements. Algorithms such as the Artificial Potential Field (APF) method and Velocity Obstacle method are widely adopted for real-time obstacle avoidance due to their simple structures and computational efficiency. Nevertheless, APF methods are prone to local minima, while VO-based approaches rely on constant-velocity assumptions of obstacles. In complex environments, both methods can easily lead to deadlock situations or oscillatory behavior near the target [7,8].

To address these limitations, numerous hybrid and improved approaches have been proposed. Yang et al. [9] proposed a Traffic-Feature Informed A-star (TFIA-star) algorithm incorporating historical AIS data to reduce computation and memory costs in large-scale path planning. Luo et al. [10] proposed an ocean-current-guided RRT* algorithm that incorporates adaptive multi-factor guidance strategies to mitigate directional uncertainty in traditional RRT under marine environments. Wang et al. [11] developed a hybrid planning method combining an improved APF with RRT, where gravitational coefficient optimization and dynamic step-size strategies were introduced to enhance global path-finding capability, while maritime rules and collision risk indices were integrated to achieve regulation-compliant dynamic obstacle avoidance.

Although the aforementioned methods have demonstrated promising performance in relatively simple scenarios, they often neglect invisible yet critical factors prevalent in real marine environments, such as nonlinear hydrodynamic resistance and ocean current disturbances. As a result, planned paths may deviate significantly during execution due to environmental forces [12], making it difficult to guarantee long-term energy efficiency and navigation stability for USV [13].

In recent years, with the rapid development of DRL, these techniques have been increasingly applied to USV navigation. DRL algorithms exhibit strong adaptability to complex, unknown, and dynamic marine environments. Algorithms such as Deep Deterministic Policy Gradient (DDPG) [14] and Twin Delayed Deep Deterministic Policy Gradient (TD3) [15] have been successfully applied to continuous control tasks. Moreover, SAC [16] enhances stochastic exploration through a maximum-entropy framework, while PPO [17] achieves improved training stability via constrained policy updates. Hierarchical DRL frameworks and hybrid combinations of DRL with traditional methods have also been widely investigated. Li et al. [18] proposed an APF-enhanced DQN method incorporating COLREGS for safe path planning. Zhang et al. [19] introduced a hierarchical neural network-based DRL approach for port environments, demonstrating strong safety performance in obstacle avoidance. Woo et al. [20] further incorporated the International Regulations for Preventing Collisions at Sea into DRL to achieve compliant collision avoidance in multi-vessel encounter scenarios. Guan et al. [21] proposed a navigation strategy combining PRM and PPO, where instantaneous risk fields were incorporated into the reward function. Xie et al. [22] developed a hybrid learning framework combining reinforcement learning and inverse control, enabling adaptive decision-making between an LSTM-based inverse model controller and a model-free A3C policy via Q-learning.

Despite these advances, the application of DRL to complex marine environments still faces several critical challenges:

1. Conventional Prioritized Experience Replay (PER) mechanisms struggle to extract highly valuable successful experiences from large volumes of failed samples, often leading to slow or unstable convergence.
2. Environmental fidelity remains insufficient, as many studies rely on simplified virtual environments or artificially synthesized current fields, limiting the transferability of learned policies to real-world scenarios [23].
3. Purely end-to-end policies exhibit blind exploration risks during early training stages, and sparse rewards further slow convergence, making it difficult to guarantee safe separation distances in environments with multiple dynamic obstacles.
4. Most path planning studies primarily focus on shortest-path generation or obstacle avoidance effectiveness, while comprehensive comparative analysis of avoidance trajectories remains largely unexplored [24].

To address the above challenges, this paper proposes an improved hybrid path planning algorithm for USV operating in complex marine environments, termed H_RS_TD3. The proposed method integrates the stability of traditional control strategies with the adaptability of reinforcement learning, aiming to tackle USV navigation problems under coupled strong ocean current disturbances and dynamic obstacle interactions. The main contributions of this paper are summarized as follows:

1. High-fidelity real-world environment reconstruction based on multi-source data: Instead of relying on synthetic virtual environments, a realistic simulation platform is constructed using GEBCO global bathymetric data and CMEMS physical reanalysis products. This platform incorporates real geographical constraints and spatiotemporally varying non-uniform ocean currents, enabling the learned policies to exhibit robustness against realistic hydrodynamic disturbances.
2. Hybrid safety-aware decision-making architecture integrating TPN and APF: To mitigate the blind exploration behavior of pure DRL during early training, a risk-aware mechanism is designed. TPN is employed to extract historical obstacle motion features and predict future trajectories, while a vectorized APF is embedded as a low-level safety constraint. This architecture enhances anticipatory dynamic obstacle avoidance while deterministically preventing extreme collision risks through repulsive force guidance.
3. CDA-PER mechanism: To improve sample efficiency under sparse reward conditions, a nonlinear priority sampling strategy jointly driven by path curvature and TD error is proposed. This mechanism automatically identifies and repeatedly emphasizes critical obstacle avoidance experiences in complex interaction scenarios, significantly accelerating convergence speed and improving policy quality.

## 2. Problem Formulation and System Modeling

### 2.1. Coordinate Systems and Kinematics

**Figure 1:**
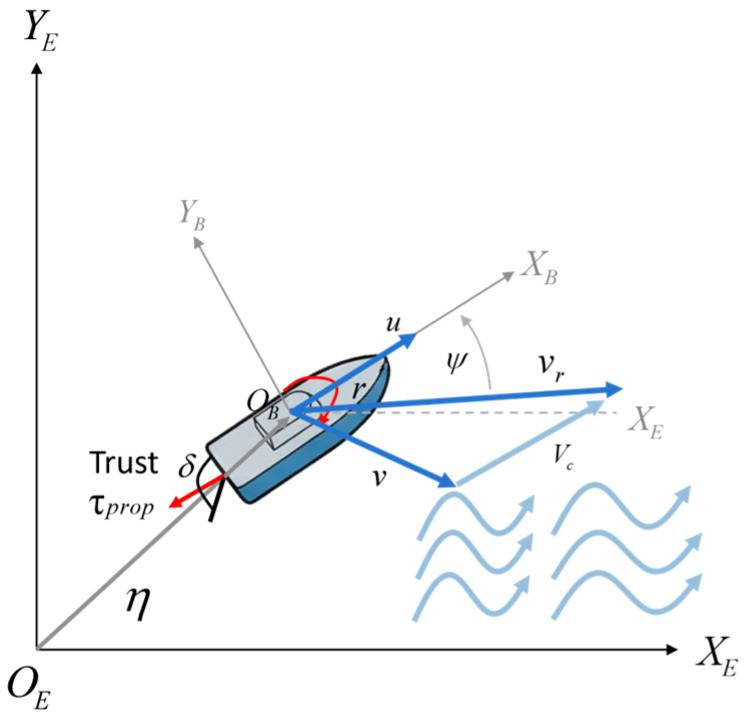
The USV motion mathematical model. Note: Blue arrows represent velocity components; red arrows denote control inputs and motion states; light blue wavy arrows indicate ocean current velocity. The inertial and body-fixed coordinate frames represent position and orientation, respectively.

As illustrated in Figure 1, the planar motion of the USV is described using an inertial coordinate frame $\{E\}-O_{E}X_{E}Y_{E}$ and a body-fixed coordinate frame $\{B\}-O_{B}X_{B}Y_{B}$. The pose vector of the USV expressed in the inertial frame {E} is defined as

(1) $$\eta ={\left[x,y,\psi \right]}^{T}$$

where x and y denote the north and east positions of the USV, respectively, and ψ represents the yaw angle.

The velocity vector expressed in the body-fixed frame {B} is given by

(2) $$\nu ={\left[u,v,r\right]}^{T}$$

where u, v and r correspond to the surge velocity, sway velocity, and yaw rate, respectively.

The transformation between the two coordinate frames is described by the rotation matrix R(ψ). Accordingly, the relationship between the inertial-frame velocity $\dot{\eta }$ and the body-frame velocity ν is expressed as

(3) $$\dot{\eta }=R(\psi )\nu$$

Expanding this relation yields

(4) $$\left[\begin{array}{c} \dot{x}\\ \dot{y}\\ \dot{\psi } \end{array}\right]=\left[\begin{array}{ccc} \cos\psi & -\sin\psi & 0\\ \sin\psi & \cos\psi & 0\\ 0 & 0 & 1 \end{array}\right]\left[\begin{array}{c} u\\ v\\ r \end{array}\right]$$

### 2.2. High-Fidelity Environmental Modeling and Dynamics

#### 2.2.1. Dynamics with Ocean Currents

Based on the marine vehicle modeling framework proposed by Fossen [25], a three-degree-of-freedom (3-DOF) dynamic model of the USV that explicitly accounts for environmental disturbances is established [26,27]. To accurately capture the physical effects of realistic ocean conditions on the vessel, the concept of relative velocity is introduced to correct the hydrodynamic damping terms.

The USV dynamics are expressed as

(5) $$M\dot{v}+C(v)v+D(v_{r})v_{r}=\tau$$

where $M\in {\mathbb{R}}^{3\times 3}$ denotes the inertia matrix, $C(v)\in {\mathbb{R}}^{3\times 3}$ is the Coriolis and centripetal matrix, $D(v_{r})\in {\mathbb{R}}^{3\times 3}$ represents the hydrodynamic damping matrix, and $\tau \in {\mathbb{R}}^{3\times 1}$ is the control input vector.

To faithfully model speed reduction under adverse currents and lateral drift effects, the relative velocity $v_{r}$ in the damping term $D(v_{r})v_{r}$ is defined as the velocity of the USV relative to the ocean current:

(6) $$\nu_{r}=\nu -R{\left(\psi \right)}^{T}V_{c}$$

where $V_{c}={\left[u_{c},v_{c},0\right]}^{T}$ denotes the ocean current velocity vector expressed in the inertial frame {E}.

By projecting the observed local ocean current into the body-fixed frame {B} through the rotation matrix $R{\left(\psi \right)}^{T}$, the proposed model is able to dynamically capture the realistic physical resistance induced by following currents, opposing currents, and lateral currents acting on the USV. This ensures the USV navigation response is based on the immediate hydrodynamic environment perceived at its current coordinates.

#### 2.2.2. Real-World Static and Dynamic Environment Reconstruction

Unlike conventional studies that rely on simplified geometric environments, this paper constructs a high-fidelity simulation environment that integrates both static maps and dynamic ocean currents.

**Figure 2:**
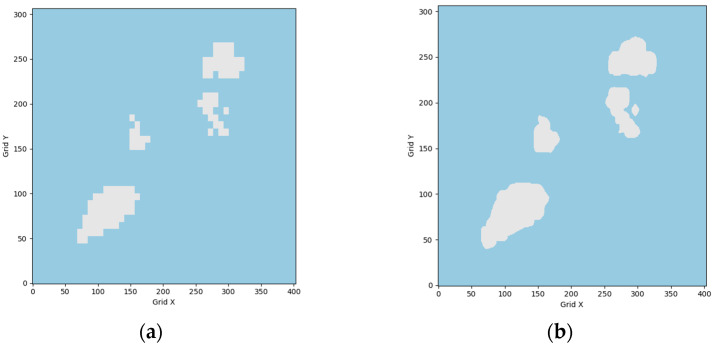
Illustration of grid-map smoothing and inflation processing: (**a**) raw grid map; (**b**) smoothed grid map. Note: Grey and blue areas represent land and ocean, respectively.

As shown in Figure 2, the static map environment is generated using GEBCO bathymetric elevation data. To mitigate the aliasing artifacts caused by direct rasterization of the raw data, this paper applies a bilinear interpolation–based smoothing correction. This procedure effectively eliminates discretization-induced geometric distortions and produces static obstacle maps with smooth boundaries that closely match real-world geographic features.

**Figure 3:**
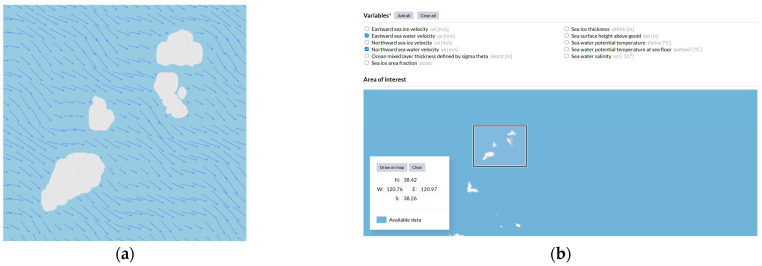
Dynamic ocean current field based on CMEMS real-world data: (**a**) visualized ocean current field; (**b**) CMEMS data selection interface. Note: Arrows in (**a**) represent current velocity vectors; the asterisk in (**b**) denotes mandatory variables.

As illustrated in Figure 3, considering that ocean current disturbances in realistic marine environments are a critical factor affecting the accuracy of USV path planning, this paper abandons simplified artificial flow-field assumptions and adopts ocean current data provided by the CMEMS Global Ocean Physics Analysis and Forecast product. A specific sea region (longitude 120.76° E–120.97° E, latitude 38.26° N–38.42° N) is selected, and the dataset includes eastward current velocity components $u_{cur}$ and northward components $v_{cur}$.

During simulation, the original NetCDF data are mapped onto a grid-based environment. The ocean current disturbance vector $V_{c}$ at any spatial location (x,y) is obtained through spatiotemporal interpolation, thereby enabling the realistic modeling of non-uniform ocean currents that vary across both time and space.

To ensure the reproducibility of the high-fidelity marine environment, the key parameters regarding the dataset construction and the USV physical simulation are summarized in Table 1.

### 2.3. MDP Formulation

The USV path planning problem under complex marine conditions is modeled in this paper as an MDP, which is defined by the tuple (S,A,P,R,γ).

Where S denotes the state space, A denotes the action space, P represents the state transition probability determined by the USV dynamics and environment interactions, R is the reward function, and γ is the discount factor.

#### 2.3.1. State Space

**Figure 4:**
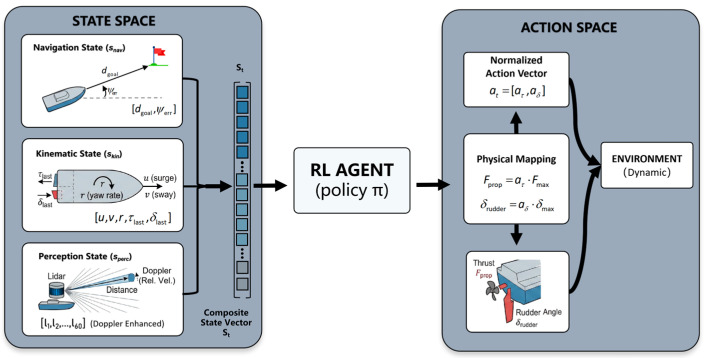
MDP-Based State and Action Space Formulation.

As shown in Figure 4, to enable accurate decision-making under partially observable environments, the state vector $s_{t}$ is designed as a composite representation that integrates goal-oriented information, the USV’s own motion states, and environmental perception. The dimensionality of the state space is set to $D_{s}=68$, and the state vector is defined as

(7) $$s_{t}=[s_{nav},s_{kin},s_{perc}]$$

1. Navigation State ($s_{nav}$)

This component describes the geometric relationship between the USV and the target, serving as high-level guidance for navigation:

(8) $$s_{nav}=[d_{goal},\psi_{err}]$$

where $d_{goal}$ denotes the Euclidean distance from the current position to the target, and $\psi_{err}$ represents the heading error, defined as the difference between the target bearing and the current heading angle. Both variables are normalized.

2. Kinematic State ($s_{kin}$)

To account for ocean current disturbances and inertial effects, the kinematic state incorporates the USV body-fixed velocities and the control inputs from the previous time step:

(9) $$s_{kin}=[u,v,r,\tau_{last},\delta_{last}]$$

where u,v are the surge and sway velocities in the body-fixed frame, r is the yaw rate, and $\tau_{last},\delta_{last}$ denote the previously applied thrust and rudder angle, respectively, ensuring control continuity.

3. Perception State ($s_{sperc}$)

This component is obtained from an onboard 2D LiDAR sensor and consists of 60 omnidirectional laser beams:

(10) $$s_{perc}=[l_{1},l_{2},\ldots ,l_{60}]$$

To enhance the prediction capability for dynamic obstacles under non-visual sensing, Doppler Effect Logic is incorporated into the LiDAR data processing. Each beam measurement $l_{i}$ encodes not only the obstacle distance but also the relative velocity projection of dynamic obstacles along the beam direction. When obstacles approach rapidly, the corresponding perception values undergo nonlinear mapping, enabling the agent to distinguish between static obstacles and dynamic threats.

#### 2.3.2. Action Space

As shown in Figure 4, considering that the USV is a typical underactuated system, the action space A is defined as a continuous control space. The action vector $a_{t}$ consists of two core control commands:

(11) $$a_{t}={\left[a_{\tau },a_{\delta }\right]}^{T},\;a_{\tau }\in [-1,1],\;a_{\delta }\in [-1,1]$$

where $a_{\tau }$ represents the thrust command controlling the main propeller output, and $a_{\delta }$ denotes the rudder angle command for heading control.

To comply with the underlying dynamics model (based on the MMG model), the normalized action outputs are mapped to physical control inputs as follows:

(12) $$\left\{\begin{array}{c} F_{prop}=a_{\tau }\cdot F_{max}\\ \delta_{rudder}=a_{\delta }\cdot \delta_{max} \end{array}\right.$$

where $F_{max}$ is the maximum effective thrust, and $\delta_{max}$ is the maximum allowable rudder angle.

This continuous action space formulation avoids control oscillations caused by discrete actions and facilitates the generation of smooth trajectories that satisfy the USV’s dynamic constraints.

#### 2.3.3. Reward Function Design

The reward function plays a critical role in determining the convergence behavior of the learning algorithm and the resulting navigation policy of the USV. As demonstrated by Zhang et al. [28], foundational reward formulations typically include an arrival reward, a distance progress reward, and a collision penalty to ensure basic navigation safety and task completion. Building upon these fundamental elements, this paper further introduces a quadratic safety potential penalty, a navigation efficiency reward, and a step-wise time penalty to simultaneously address task efficiency and navigation safety in complex marine environments. Consequently, the instantaneous reward is defined as the weighted sum of these six components. This composite reward structure jointly promotes rapid task completion, safe obstacle avoidance, and kinematically efficient navigation.

The overall reward at time step t t is formulated as

(13) $$R_{t}=r_{arrival}+r_{dist}+r_{collision}+r_{safety}+r_{nav}+r_{step}$$

when the distance between the USV and the target satisfies Equation (14), the USV is considered to have reached the goal. In this case, a one-time arrival reward is granted, and the current episode is terminated. This large positive reward provides a clear “task completion” signal to the agent and accelerates policy convergence:

(14) $$d_{t}=\|p_{t}-p_{goal}\|<d_{th}(d_{th}=0.5\;m)$$

where $d_{th}$ is the threshold distance. It is empirically set to 0.5 to provide spatial tolerance and prevent policy oscillation.

(15) $$r_{\mathrm{arrival}}=+R_{\max}\;(R_{\max}=10.0)$$

where $R_{max}$ is the maximum overall reward. It is set to 10.0 to dominate step rewards and ensure goal-reaching is the absolute priority.

To encourage rapid progress toward the target, a distance-based progress reward $r_{\mathrm{dist}}$ is introduced:

(16) $$r_{\mathrm{dist}}=k_{p}(d_{t-1}-d_{t})$$

where $d_{t-1}$ and $d_{t}$ denote the target distances before and after the current maneuver, respectively. The scaling factor $k_{p}$ amplifies the effect of distance reduction and strengthens the convergence tendency. $k_{p}$ is empirically set to 0.1. This bounds the dense reward and prevents reward hacking, such as exploiting cyclic motions.

To incentivize effective obstacle avoidance and ensure navigation safety, a severe collision penalty $r_{\mathrm{collision}}$ is imposed when a collision with static terrain or dynamic obstacles is detected:

(17) $$r_{\mathrm{collision}}=\left\{\begin{array}{cc} -R_{c}, & \mathrm{if}\;\mathrm{collision}\;\mathrm{occurs}\\ 0, & \mathrm{otherwise} \end{array}\right.$$

where $R_{c}$ represents the collision cost. $R_{c}$ is symmetrically set to 10.0 to strictly enforce collision avoidance.

To prevent the USV from approaching obstacles excessively before a collision occurs, a quadratic safety potential field penalty $r_{\mathrm{safety}}$ is constructed within a predefined safety radius:

(18) $$r_{\mathrm{safety}}=\left\{\begin{array}{cc} -\lambda_{s}{\left(\frac{d_{\mathrm{safe}}-d_{\min}}{d_{\mathrm{safe}}}\right)}^{2}, & d_{\min}<d_{\mathrm{safe}}\\ 0, & \mathrm{otherwise} \end{array}\right.\;$$

where $d_{\min}$ denotes the minimum obstacle distance measured by the lidar, $d_{\mathrm{safe}}=3.0\;m$ defines the safety buffer zone, and $\lambda_{s}=0.5$ is a scaling factor. Unlike linear penalties, the quadratic formulation imposes rapidly increasing costs as the obstacle distance approaches zero, effectively pushing the agent away from high-risk regions.

To optimize kinematic performance and prevent stagnation, a navigation efficiency reward $r_{\mathrm{nav}}$ is designed based on the surge velocity u and heading error $\theta_{\mathrm{err}}$:

(19) $$r_{\mathrm{nav}}=\left\{\begin{array}{cc} \alpha \cdot u\cdot \cos(\theta_{\mathrm{err}}), & \mathrm{if}\;\cos(\theta_{\mathrm{err}})>0\\ -\beta , & \mathrm{if}\;0\leq u<u_{\mathrm{th}}\\ -2\beta , & \mathrm{if}\;u<0 \end{array}\right.$$

where α=0.01 rewards fast motion toward the target direction, while β=0.05 and $u_{\mathrm{th}}=0.1\;m/s$ penalize low-speed cruising and reverse motion, thereby encouraging sustained forward navigation.

Finally, a constant step-wise penalty is applied at each time step:

(20) $$r_{\mathrm{step}}=-0.05$$

## 3. Conventional TD3 Algorithm

**Figure 5:**
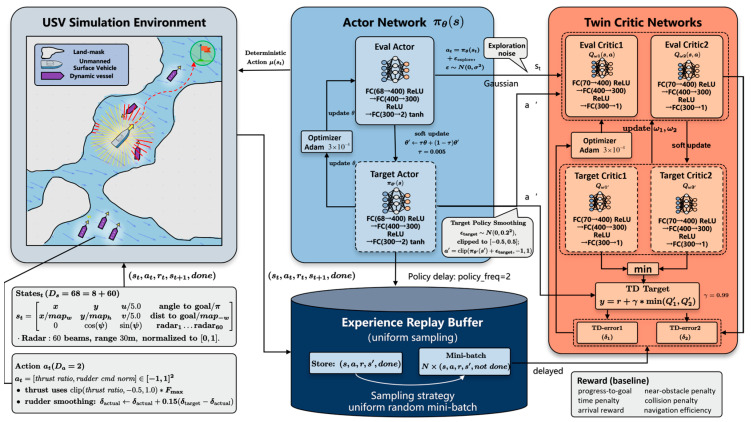
TD3 Network Architecture.

The TD3 algorithm [29] adopts an Actor–Critic–based delayed deterministic policy gradient framework. By integrating clipped double Q-learning, delayed policy updates, and target policy smoothing, TD3 effectively alleviates the overestimation problem commonly observed in value function learning [30]. As shown in Figure 5, the overall architecture consists of two main components, namely the policy network (Actor) and the value networks (Critics), forming a total of six deep neural networks:

1. Actor Network (policy network π)

The Actor network is responsible for approximating a deterministic policy function $\mu (s|\theta^{\mu })$, which maps the current system state to continuous control actions. Specifically, it takes a 68-dimensional state vector $s_{t}$, which includes navigation information, motion states, and radar perception features, as input. After feature extraction through multiple fully connected layers, the network outputs a normalized action vector $a_{t}$ within the range [−1, 1] via a Tanh activation function. The action vector consists of continuous control commands for propulsion and rudder angle.

2. Twin Critic Networks (value networks $Q_{1},Q_{2}$)

To address the Q-value overestimation issue present in conventional DDPG algorithms [31], TD3 introduces two Critic networks with identical architectures but independent parameters. Each Critic takes the concatenated state–action pair $\left(s_{t},a_{t}\right)$ as input and independently estimates the corresponding action-value function $Q(s_{t},a_{t})$. When computing the target Q-value, TD3 selects the minimum value predicted by the two Critic networks:

(21) $$y_{t}=r_{t}+\gamma \min_{i=1,2}\;Q_{i^{\prime }}(s_{t+1},\pi^{\prime }(s_{t+1})+\epsilon )$$

where $y_{t}$ is the target Q-value, $r_{t}$ is the immediate reward, γ is the discount factor, $Q_{i^{\prime }}$ (i=1,2) denotes the two target Critic networks, $s_{t+1}$ is the next state, $\pi^{\prime }$ represents the target Actor network, and ϵ denotes the clipped exploration noise added to the target action.

This clipped double Q-learning mechanism significantly improves the stability of value estimation.

3. Target Networks

Both the Actor and the Twin Critic networks are associated with corresponding target networks $\left(\pi^{\prime },Q_{1^{\prime }},Q_{2^{\prime }}\right)$. The parameters of the target networks are updated through a soft update strategy governed by a soft update coefficient τ. Setting this coefficient to a value much less than 1 (τ≪1) ensures a strictly slow parameter update rate. This slow updating process allows them to lag behind the main networks and further suppresses training oscillations.

Although the standard TD3 algorithm demonstrates strong performance in generic continuous control tasks, directly applying it to complex USV dynamic path planning scenarios still exposes several critical limitations, including delayed environmental perception, overly aggressive exploration behavior, and low sample utilization efficiency. These issues severely restrict its engineering applicability under realistic marine conditions.

## 4. Improved H_RS_TD3 Algorithm Design

**Figure 6:**
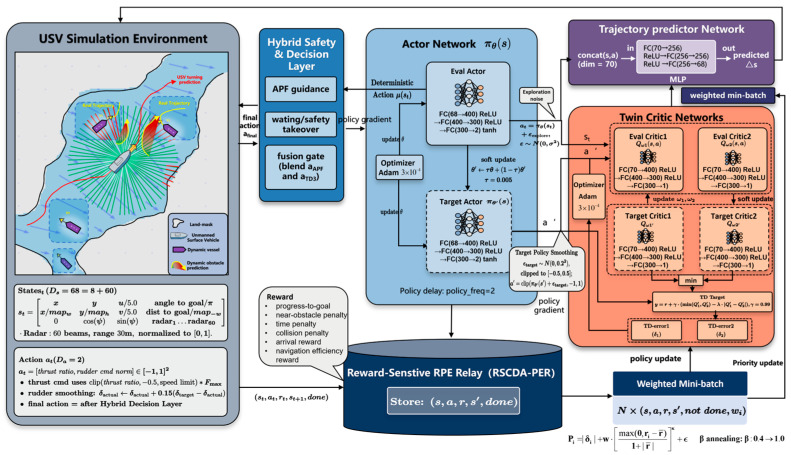
H_RS_TD3 Network Architecture.

Figure 6 illustrates the overall architecture of the proposed H_RS_TD3 framework. To address the key limitations of standard TD3 when deployed in realistic marine environments—including perceptual delay, lack of explicit safety guarantees, and inefficient utilization of interaction data—this study proposes an improved deep reinforcement learning architecture that integrates trajectory prediction with hybrid potential-field-based safety constraints. The proposed framework adopts a modular and cooperative design philosophy, embedding perception enhancement, safety-aware decision fusion, and robust value learning into a unified closed-loop interaction process.

The overall operation of H_RS_TD3 can be conceptually divided into three sequential yet tightly coupled phases: predictive perception, constrained decision-making, and robust policy optimization.

### 4.1. Hybrid Safety Decision

**Figure 7:**
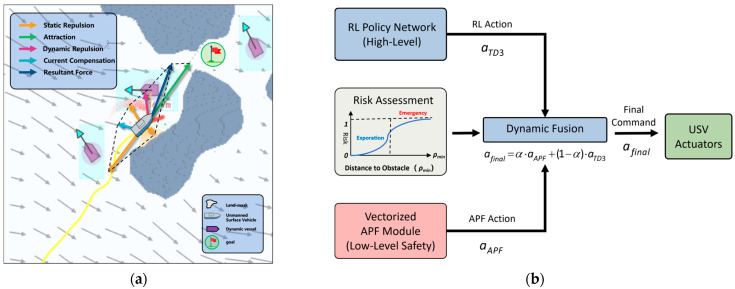
Hybrid Safety Decision Diagram: (**a**) vectorized force analysis diagram; (**b**) risk-aware dynamic fusion architecture diagram.

Figure 7 illustrates the proposed Hybrid Safety and Decision Layer, which is designed to balance the strong local safety guarantees of classical methods with the long-horizon planning capability of reinforcement learning. To this end, a risk-aware hybrid decision architecture is constructed, consisting of two parallel computational modules: a vectorized artificial potential field (APF) that provides low-level safety guidance, and the H_RS_TD3 policy network, which generates high-level global decisions. These two components are integrated through a dynamic fusion mechanism, enabling a smooth transition from efficient exploration to emergency avoidance.

This hybrid design effectively addresses two well-known limitations: the tendency of conventional APF methods to fall into local minima, and the lack of safety guarantees during early exploration stages of reinforcement learning.

#### 4.1.1. Vectorized Potential Field Construction

As illustrated in Figure 7a, an enhanced vectorized artificial potential field is constructed to quantify environmental risk and enable dynamic safety intervention. Unlike traditional APF formulations that compute repulsive forces point-by-point, the proposed method employs a vectorized implementation to parallelize force computation over the entire grid map, thereby satisfying real-time requirements in complex environments.

The resultant force acting on the USV is defined as a linear superposition of multiple force components:

(22) $$F_{total}=F_{att}+\sum F_{static}+\sum F_{dyn}+F_{wall}+F_{curr}$$

where $F_{att}$ denotes the attractive force toward the goal, $F_{static}$ and $F_{dyn}$ represent repulsive forces induced by static and dynamic obstacles, respectively, $F_{wall}$ corresponds to boundary repulsion, and $F_{curr}$ is the current-compensation force.

For static obstacles, a standard APF-based repulsive formulation is adopted. Let $\eta_{static}$ denote the repulsion gain and $d_{0,static}$ the safety influence radius. The repulsive force is defined as

(23) $$F_{static}=\left\{\begin{array}{cc} \eta_{static}\cdot {\left(\frac{1}{d(q,O_{i})}-\frac{1}{d_{0,static}}\right)}^{2}\cdot \frac{{\vec{n}}_{io}}{d{\left(q,O_{i}\right)}^{2}}, & if\;0<d(q,O_{i})\leq d_{0,static}\\ 0, & if\;d(q,O_{i})>d_{0,static} \end{array}\right.$$

To account for the increased uncertainty associated with moving obstacles, a dynamic inflation mechanism is introduced to construct a wider risk buffer. In implementation, the repulsion gain and influence radius are scaled as $\eta_{dyn}=2.5\times \eta_{static}$ and $d_{0,dyn}=3.0\times d_{0,static}$, yielding the following repulsive force:

(24) $$F_{dyn}=\left\{\begin{array}{cc} 2.5\eta_{static}\cdot {\left(\frac{1}{d(q,O_{i})}-\frac{1}{3.0d_{0,static}}\right)}^{2}\cdot \frac{{\vec{n}}_{io}}{d{\left(q,O_{i}\right)}^{2}}, & if\;0<d(q,O_{i})\leq 3.0d_{0,static}\\ 0, & if\;d(q,O_{i})>3.0d_{0,static} \end{array}\right.$$

where $d(q,O_{i})$ denotes the Euclidean distance between the current USV position q and the closest point on the surface of obstacle $O_{i}$, ${\vec{n}}_{io}$ is the unit vector pointing from the obstacle toward the USV, $\eta_{static}$ and $d_{0,static}$, static represent the baseline repulsion gain and the safety distance threshold in static environments, respectively.

To mitigate drift induced by strong ocean currents, a current-compensation force $F_{curr}$ is introduced. This force is proportional to the estimated local flow velocity vector ${\hat{V}}_{c}$ but acts in the opposite direction:

(25) $$F_{curr}=-\zeta \cdot {\hat{V}}_{c}$$

where ζ is a tunable gain controlling the strength of current compensation, and ${\hat{V}}_{c}$ denotes the local current velocity estimated by onboard sensors.

Furthermore, to prevent boundary collisions under strong disturbances, a nonlinear wall-repulsion force $F_{wall}$ is defined. When the distance $\rho_{\mathrm{wall}}$ between the USV and the nearest shoreline falls below a predefined threshold $\rho_{safe}$, an exponentially increasing repulsive force is activated:

(26) $$F_{wall}=\left\{\begin{array}{cc} k_{wall}\cdot \exp(-\rho_{wall})\cdot n_{wall}, & if\;\rho_{wall}<\rho_{safe}\\ 0, & otherwise \end{array}\right.$$

where $\rho_{wall}$ denotes the outward normal vector of the boundary.

#### 4.1.2. Risk-Aware Dynamic Fusion Mechanism

To enable adaptive switching between efficient exploration and safety-critical control, a risk-aware dynamic fusion mechanism is introduced, as shown in Figure 7b. Rather than relying on a single distance threshold, this mechanism defines an Environmental Risk Coefficient α based on multi-source perception, which is dynamically adjusted according to the minimum detected obstacle distance $\rho_{min}$.

The fusion coefficient α is computed using a modified sigmoid function:

(27) $$\alpha =1-\frac{1}{1+\exp(-k_{fuse}\cdot (\rho_{min}-d_{crit}))}$$

where $d_{crit}$ denotes the critical safety distance and $k_{fuse}$ controls the smoothness of the transition.

Based on the value of $\rho_{min}$, the system operates in two distinct modes:

1. Safety Takeover Mode ($\rho_{min}<d_{crit}$): α→1, and the APF component dominates the control action, enforcing conservative avoidance to ensure low-level safety.
2. Efficient Exploration Mode ($\rho_{min}>d_{crit}$): α→0, and the reinforcement learning policy assumes primary control authority, focusing on path optimization and efficiency.

The final action command delivered to the USV dynamics is obtained via linear blending:

(28) $$a_{final}=\alpha \cdot a_{APF}+(1-\alpha )\cdot a_{TD3}$$

Through this fusion strategy, the proposed framework maintains the generalization capability of TD3 in complex flow environments while using APF-based safety constraints to suppress unsafe actions.

### 4.2. TPN Module

**Figure 8:**
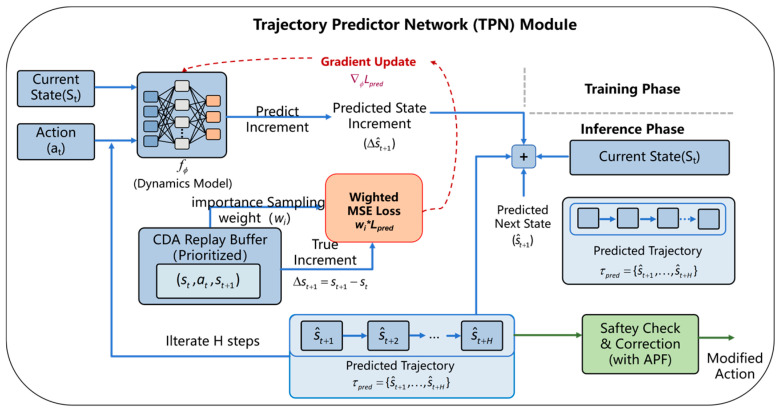
Trajectory Prediction Network Architecture.

Figure 8 illustrates the architecture of the TPN, which is integrated in parallel with the Actor-Critic framework to alleviate the impact of partial observability in complex environments. Unlike conventional model-free approaches, the TPN explicitly learns the environment transition manifold, enabling the USV to anticipate future states before executing actions.

The TPN is parameterized by ϕ and represents a deterministic transition model. To improve numerical stability and accelerate convergence, the network predicts the state increment rather than the absolute next state. Given the current state $s_{t}$ and the action $a_{t}$ generated by the policy, the prediction process is formulated as

(29) $$\Delta {\hat{s}}_{t+1}=f_{\phi }(s_{t},a_{t}),\;{\hat{s}}_{t+1}=s_{t}+\Delta {\hat{s}}_{t+1}$$

where $f_{\phi }$ denotes the dynamics mapping function parameterized by ϕ, $s_{t}$ is the system state vector at time step t, $a_{t}$ is the action output by the policy network, $\Delta {\hat{s}}_{t+1}$ and $\;{\hat{s}}_{t+1}$ represent the predicted state increment and the predicted next state, respectively.

During training, the TPN is optimized via an auxiliary supervised regression task based on the PER mechanism. Specifically, the mean squared error (MSE) between the predicted state increment and the true transition tuple $\left(s_{t},a_{t},s_{t+1}\right)$ sampled from the replay buffer D is minimized. The loss function is defined as

(30) $$L_{TPN}(\phi )=E_{(s_{t},a_{t},s_{t+1},w_{i})\sim D}\left[w_{i}\cdot ||(s_{t+1}-s_{t})-f_{\phi }(s_{t},a_{t})|{|}_{2}^{2}\right]$$

where $w_{i}$ denotes the importance sampling weight. As an auxiliary differentiable dynamics module, the TPN captures latent physical properties such as USV inertia and hydrodynamic effects under ocean currents, thereby regularizing the representation learning of the main policy network.

Specifically, the TPN is trained online by sampling transition tuples $\left(s_{t},a_{t},s_{t+1}\right)$ directly from the replay buffer D, which eliminates the need for any pre-collected offline datasets.

**Figure 9:**
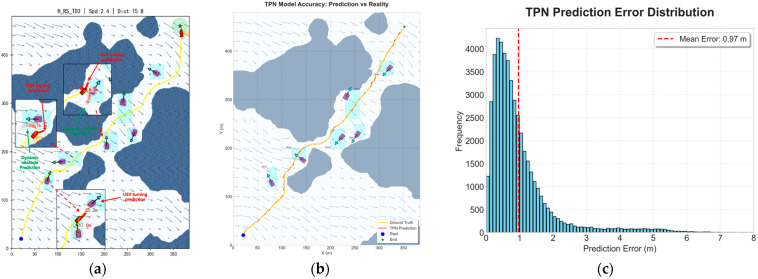
Trajectory prediction process and performance evaluation: (**a**) real-time trajectory prediction diagram; (**b**) trajectory comparison between the TPN prediction and the ground truth; (**c**) prediction error distribution of the TPN.

Figure 9a visualizes the trajectory prediction process. The proposed method adopts a dual-stream prediction mechanism. Leveraging the learned dynamics features, the TPN performs an H-step rolling horizon inference during navigation and recursively feeds the predicted states back into the network, generating a short-term predicted trajectory:

(31) $$\tau_{pred}=\{{\hat{s}}_{t+1},\ldots ,{\hat{s}}_{t+H}\}$$

The horizon H dictates the USV’s look-ahead depth. Since the trajectory is generated via H sequential forward passes, the computational load scales linearly with H, directly influencing the Infer Time metric.

To validate the reliability of the TPN, the present study analyzed its prediction performance in complex marine environments. As shown in Figure 9b, the continuous predicted trajectory (red line) closely aligns with the actual ground-truth trajectory of the USV (yellow line). Furthermore, Figure 9c presents the prediction error distribution, revealing that the TPN achieves highly accurate state estimations with an overall mean prediction error of only 0.97 m.

During the rollout process, radar-based observations are processed simultaneously. Based on a short-term constant-velocity model, the kinematic projection of dynamic obstacles is computed to estimate their future trajectories:

(32) $$\tau_{obs}^{k}=\{p_{obs,t+1}^{k},p_{obs,t+2}^{k},\ldots ,p_{obs,t+H}^{k}\}$$

where $\tau_{obs}^{k}$ denotes the predicted trajectory set of the k-th dynamic obstacle, and $p_{obs,t+H}^{k}$ represents the estimated spatial position of that obstacle at time t+H.

By evaluating the potential spatiotemporal intersections between the predicted USV trajectory $\tau_{pred}$ and the projected obstacle trajectories $\tau_{obs}$, the module can identify dynamic collision risks in advance.

This mechanism provides the system with a critical safety buffer, enabling the USV to proactively avoid moving threats based on anticipated future situations, rather than reacting solely to instantaneous distance measurements.

### 4.3. CDA-PER Mechanism

Conventional PER typically relies on linear sampling based on the TD error. This strategy may overlook samples with small TD errors that are nevertheless critical for task completion. This issue is particularly evident in highly dynamic USV obstacle avoidance tasks with sparse rewards, where simple linear reward weighting fails to distinguish a small number of high-value samples, such as successful extreme avoidance maneuvers, from a large number of mediocre experiences.

**Figure 10:**
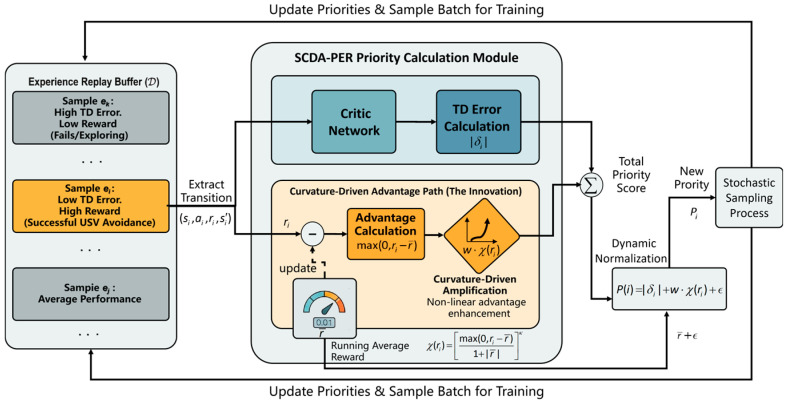
Dual-stream priority computation architecture.

To address this limitation, a CDA-PER mechanism is proposed. As shown in Figure 10, it adopts a dual-stream priority computation architecture. In addition to the TD-error-based priority computed by the critic network, a parallel curvature-driven advantage path is introduced. By incorporating a nonlinear advantage amplification exponent γ together with a dynamic normalization factor, the replay priority is reformulated as

(33) $$P_{i}=|\delta_{i}|+w\cdot {\left[\frac{\max(0,r_{i}-\bar{r})}{1+|\bar{r}|}\right]}^{\kappa }+\epsilon$$

where $\left|\delta_{i}\right|$ denotes the absolute TD error, w is the weighting coefficient of the advantage term, $r_{i}$ represents the instantaneous reward of the current sample, and $\bar{r}$ denotes the running average reward. The constant ϵ guarantees a non-zero sampling probability for all transitions and preserves basic exploration.

The denominator $1+|\bar{r}|$ enables dynamic normalization across different reward scales and improves robustness under varying environmental reward magnitudes. Meanwhile, the numerator term $\max(0,r_{i}-\bar{r})$, combined with the exponent κ, provides nonlinear amplification for samples whose rewards exceed the average level. This mechanism ensures that critical success experiences, particularly those corresponding to successful obstacle avoidance, are preferentially replayed during training.

### 4.4. Uncertainty-Aware Conservative Q-Learning Mechanism

The standard TD3 algorithm mitigates Q-value overestimation by adopting clipped double Q-learning, where the minimum value of two target critic networks is used. However, in complex marine environments characterized by sparse rewards and highly dynamic obstacles, USVs frequently encounter out-of-distribution states. Under such conditions, both critic networks may produce unreliable and overly optimistic value estimates. To further enhance safety and robustness under epistemic uncertainty, an uncertainty-aware conservative Q-learning mechanism is introduced into the critic target update. This design effectively buffers the strategy against unmodeled vessel dynamics and stochastic environmental forces, providing a safety margin when the USV’s motion response deviates from expectations.

The disagreement between the two target critic networks is used as a proxy for value estimation uncertainty, defined as

(34) $$U(s^{\prime },a^{\prime })=|{Q^{\prime }}_{\theta_{1}^{\prime }}(s^{\prime },a^{\prime })-{Q^{\prime }}_{\theta_{2}^{\prime }}(s^{\prime },a^{\prime })|$$

where ${Q^{\prime }}_{\theta_{1}^{\prime }}(s^{\prime },a^{\prime })$ and ${Q^{\prime }}_{\theta_{2}^{\prime }}(s^{\prime },a^{\prime })$ represent the action-value estimates output by the two target critic networks, respectively.

A larger disagreement indicates higher uncertainty in the value estimation for the current state-action pair. Based on this uncertainty measure, a more conservative target Q-value is constructed by penalizing the standard minimum Q-value with an uncertainty-weighted term:

(35) $${Q^{\prime }}_{conservative}(s^{\prime },a^{\prime })=\min_{i=1,2}{Q^{\prime }}_{\theta_{i}^{\prime }}(s^{\prime },a^{\prime })-\lambda_{u}\cdot U(s^{\prime },a^{\prime })$$

where $\min_{i=1,2}{Q^{\prime }}_{\theta_{i}^{\prime }}(s^{\prime },a^{\prime })$ denotes the standard minimum target Q-value used to reduce overestimation, and $\lambda_{u}>0$ is the uncertainty aversion coefficient controlling the penalty strength.

Accordingly, the final TD target for critic training is computed as

(36) $$y_{t}=r(s_{t},a_{t})+\gamma \cdot (1-d_{t})\cdot \left[\min_{i=1,2}{Q^{\prime }}_{\theta_{i}^{\prime }}({s^{\prime }}_{t+1},{a^{\prime }}_{t+1})-\lambda_{u}\cdot \left|{Q^{\prime }}_{\theta_{1}^{\prime }}({s^{\prime }}_{t+1},{a^{\prime }}_{t+1})-{Q^{\prime }}_{\theta_{2}^{\prime }}({s^{\prime }}_{t+1},{a^{\prime }}_{t+1})\right|\right]$$

where $r(s_{t},a_{t})$ denotes the immediate reward received from the environment, γ represents the discount factor, and $d_{t}$ is the terminal flag indicating the end of an episode.

This mechanism effectively suppresses value overestimation in highly uncertain state-action regions and encourages the agent to adopt a conservative policy. By avoiding potentially hazardous regions that are insufficiently covered by past experience, the proposed approach significantly improves navigation safety in complex and uncertain marine environments.

### 4.5. Algorithm Summary and Implementation Details

Algorithm 1 summarizes the training and execution workflow of H_RS_TD3. The process consists of four phases: predictive perception, risk-aware action fusion, environment interaction, and policy optimization. During execution, the control authority is dynamically allocated between APF and the RL policy via α (Equation (28)). During optimization, TPN is trained online using $\Delta {\hat{s}}_{t+1}$ (Equation (30)). Meanwhile, the Critics are updated with an uncertainty-aware target (Equation (36)), and high-value samples are prioritized by CDA-PER (Equation (33)). This design ensures both stable convergence and real-time performance.
**Algorithm 1: The Training and Execution Process of H_RS_TD3.**
**Input:** Max episodes M, Max steps T, prediction horizon H, soft update rate
τ
$,\;\mathrm{penalty}\;\lambda_{u}$

**Output:** Trained policy network
$\pi_{\theta }$, Trained value networks
$Q_{\theta_{1}}$
$,\;Q_{\theta_{2}}$
1$\mathrm{Initialize}\;\pi_{\theta }$$,Q_{\theta_{1}}$$,\;Q_{\theta_{2}}$$,\;f_{\phi }$$,\;\mathrm{target}\;\mathrm{networks}\;(\theta^{\prime }\leftarrow \theta$$,{\theta^{\prime }}_{1,2}\leftarrow \theta_{1,2}$), and CDA-PER buffer D;2for episode e=1,
…,
M **do**3$\;\;\;\mathrm{Reset}\;\mathrm{marine}\;\mathrm{environment}\;\mathrm{and}\;\mathrm{observe}\;\mathrm{initial}\;\mathrm{state}\;s_{0}$;4   for step t=0,
…, T−1 **do**5       **Phase 1 & 2: Predictive Perception and Action Fusion;**6$\;\;\;\;\;\;\mathrm{Predict}\;\Delta {\hat{s}}_{t+1}\leftarrow f_{\phi }(s_{t},a_{t})$ and compute risk coefficient α (Equations (27) and (29));7$\;\;\;\;\;\;\mathrm{Obtain}\;\mathrm{RL}\;\mathrm{action}\;a_{TD3}\leftarrow \pi_{\theta }(s_{t})+\epsilon_{explore}$ $\mathrm{and}\;\mathrm{APF}\;\mathrm{command}\;a_{APF}$ (Equation (25));8$\;\;\;\;\;\;\mathrm{Execute}\;\mathrm{hybrid}\;\mathrm{action}\;a_{final}\leftarrow \alpha \cdot a_{APF}+(1-\alpha )\cdot a_{TD3}$ (Equation (28));9       **Phase 3: Environment Interaction;**10$\;\;\;\;\;\;\mathrm{Execute}\;a_{final}$$,\;\mathrm{observe}\;r_{t}$$,\;\mathrm{next}\;\mathrm{state}\;s_{t+1}$$,\;\mathrm{terminal}\;\mathrm{flag}\;d_{t}$, and store in D;11       **Phase 4: Robust Policy Optimization;**12       if D contains sufficient samples **then**13      Sample a batch of N
$\mathrm{transitions}\;\mathrm{and}\;\mathrm{priority}\;\mathrm{weights}\;w_{i}$ from D;14      Update TPN ϕ
$\mathrm{by}\;\mathrm{minimizing}\;\mathrm{weighted}\;\mathrm{MSE}\;\mathrm{loss}\;L_{TPN}(\phi )$ (Equation (30));15      $\mathrm{Compute}\;\mathrm{value}\;\mathrm{uncertainty}\;U(s_{t+1},{a^{\prime }}_{t+1})$
$\mathrm{and}\;\mathrm{conservative}\;\mathrm{target}\;y_{t}$ (Equations (34) and (36));16      $\mathrm{Update}\;\mathrm{Critics}\;\theta_{1}$
$,\theta_{2}$
$\mathrm{by}\;\mathrm{minimizing}\;\mathrm{weighted}\;\mathrm{Smooth}\;L1\;\mathrm{loss}\;w.r.t\;y_{t}$;17      $\mathrm{Update}\;\mathrm{transition}\;\mathrm{priorities}\;P_{i}$ in D via CDA-PER mechanism (Equation (33));18      if t
$\left(\mathrm{mod}d_{freq}\right)$ == 0 **then**19      Update Actor θ via DPG and soft update all target networks;20$\;\mathrm{return}\;\pi_{\theta }$$,Q_{\theta_{1}}$$,\;Q_{\theta_{2}}$

Furthermore, the detailed hyperparameters utilized for the proposed H_RS_TD3 framework are explicitly listed in Table 2, ensuring full algorithmic reproducibility.

## 5. Simulation Experiments and Analysis

To comprehensively evaluate the overall performance of the proposed H_RS_TD3 algorithm in complex marine environments, a series of progressive experimental scenarios is designed. Su et al. [32] tested AUVs against 3D helical currents and dynamic obstacles. They assessed metrics including path length, inference time, and obstacle clearance. However, USVs face unique surface-level challenges. These include constrained island waterways, realistic surface ocean currents, and dense multi-vessel traffic. Inspired by this evaluation logic, we designed specific scenarios tailored for USVs. The proposed method is compared with classical planning algorithms, including A*, RRT*, and APF, and representative deep reinforcement learning algorithms, namely DDPG, TD3, and RS_TD3 (Linear PER). The experimental scenarios cover the following cases:

1. Static map: Two maps are constructed to evaluate the basic path planning capability. These simulate narrow constrained passages and sparse island topologies.
2. Static map with ocean current interference: Real ocean current fields from CMEMS are superimposed to assess robustness against flow disturbances.
3. Dynamic obstacles map: Scenarios with 2, 4, and 6 dynamic obstacles are designed to evaluate dynamic obstacle avoidance performance.
4. Dynamic obstacles map with ocean current interference: Extreme conditions combining multiple dynamic obstacles and strong ocean currents are simulated to verify overall robustness.

To quantitatively evaluate algorithm performance, three representative metrics are selected as evaluation criteria:

1. Path Length (m): This metric reflects the optimality of the planned path.
2. Infer Time (s): Inference time measures the real-time computational efficiency of the algorithm.
3. Mean obstacle clearance (MOC/m): This metric reflects the minimum safety margin between the planned path and both dynamic and static obstacles.

These metrics jointly reflect efficiency, safety, and real-time feasibility. By adapting these evaluation standards to the specific USV configurations, this paper aims to verify whether the proposed algorithm overcomes the challenges of real-time collision avoidance under coupled hydrodynamic disturbances.

All simulations were performed on a workstation, the hardware specifications of which are detailed in Table 3.

### 5.1. Static Map Experiments

**Figure 11:**
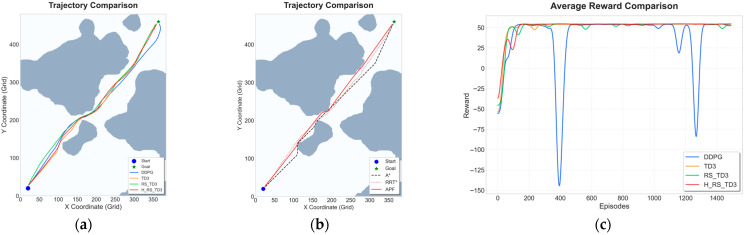
Trajectory and reward comparison on static Map 1: (**a**) trajectory comparison of reinforcement learning–based algorithms; (**b**) trajectory comparison of traditional algorithms; (**c**) reward comparison on static Map 1.

**Figure 12:**
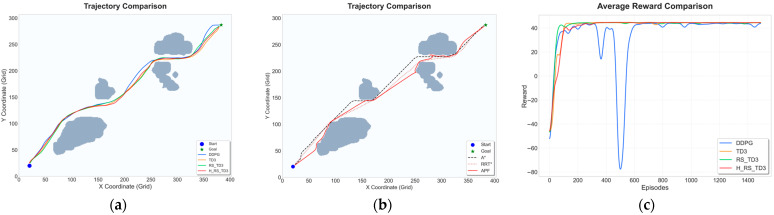
Trajectory and reward comparison on static Map 2: (**a**) trajectory comparison of reinforcement learning–based algorithms; (**b**) trajectory comparison of traditional reinforcement algorithms; (**c**) reward comparison on static Map 2.

To evaluate the fundamental path planning capability and convergence characteristics of the proposed H_RS_TD3 algorithm, comparative experiments were conducted on two static grid maps with different spatial complexities: Map 1, representing a narrow waterway with constrained passages, and Map 2, featuring relatively sparse obstacles. The quantitative results and visual performance comparisons for these scenarios are summarized in Table 4 and Table 5 and Figure 11 and Figure 12.

In the narrow Map 1 scenario, H_RS_TD3 demonstrates superior path optimality and safety. As shown in Figure 11a,b, the generated trajectory remains smooth and closely follows the global trends of A* and RRT*, effectively suppressing the oscillations observed in DDPG. Quantitative results in Table 4 show that H_RS_TD3 achieves the best path length (564.17 m) among learning-based methods while maintaining a reliable MOC of 5.77 m. Computationally, the algorithm achieves a single-step decision latency of 0.0173 s, which is significantly more efficient than the over 10 s required by traditional search planners. Finally, the reward curve in Figure 11c illustrates that H_RS_TD3 converges smoothly with minimal variance, avoiding the stability issues and performance collapses found in DDPG.

In the sparser Map 2 scenario, H_RS_TD3 demonstrates superior navigation efficiency and safety while maintaining a smooth trajectory profile. As illustrated in Figure 12a,b, the proposed method avoids the unnecessary zigzag motions seen in baseline algorithms and produces a more direct path to the goal. According to Table 5, the average path length (467.52 ± 0.71 m) remains highly competitive with traditional planners like RRT* (464.69 m), while the inference time (0.0180 s) is several orders of magnitude faster than A* and RRT*. From a safety standpoint, H_RS_TD3 maintains the largest MOC of 4.14 ± 0.36 m, significantly exceeding those of the baseline methods. Finally, the reward convergence curve in Figure 12c confirms the stability of the algorithm, showing a smoother learning process and lower variance compared to the pronounced oscillations and performance collapses of DDPG.

### 5.2. Static Map Experiments with Ocean Current Interference

**Figure 13:**
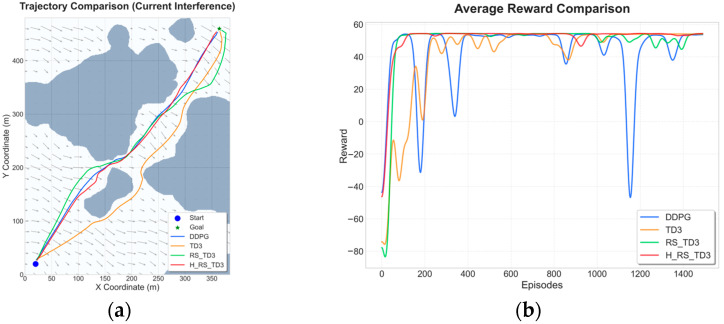
Trajectory and reward comparison on static Map 1 with ocean currents: (**a**) trajectory comparison on static Map 1 with ocean currents; (**b**) reward comparison on static Map 1 with ocean currents.

**Figure 14:**
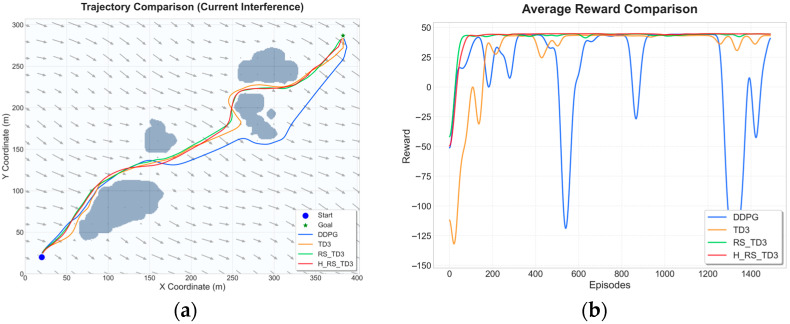
Trajectory and reward comparison on static Map 2 with ocean currents: (**a**) trajectory comparison on static Map 2 with ocean currents; (**b**) reward comparison on static Map 2 with ocean currents.

To evaluate robustness under environmental disturbances, non-uniform and time-varying ocean currents from CMEMS data were superimposed onto the static maps. This section focuses on comparing learning-based methods, as classical planners are not designed to handle such hydrodynamic dynamics. The quantitative results and visual performance comparisons for these scenarios are summarized in Table 6 and Table 7 and Figure 13 and Figure 14.

In Map 1, under current interference, H_RS_TD3 demonstrates superior anticipatory behavior and robustness. As shown in Figure 13a, the algorithm maintains a compact trajectory aligned with the navigable corridor, while standard TD3 suffers from frequent heading corrections and drift-induced detours. Quantitative data in Table 6 confirms that H_RS_TD3 achieves the most efficient path length (566.7 ± 3.40 m) and the highest MOC (5.10 ± 0.16 m). Despite the added complexity of current compensation, it preserves real-time feasibility with a stable inference time of 0.057 s. Furthermore, the reward curves in Figure 13b illustrate that H_RS_TD3 converges smoothly to a high reward level, whereas DDPG and TD3 exhibit significant performance degradation and oscillations due to flow disturbances.

In the sparser Map 2 scenario under current interference, H_RS_TD3 continues to outperform baseline methods in terms of efficiency and safety. As illustrated in Figure 14a, the proposed method effectively counteracts lateral current disturbances to maintain a smooth path, whereas DDPG adopts a less efficient detour strategy. According to Table 7, H_RS_TD3 attains the shortest average path length (468.3 ± 1.32 m) and the fastest inference speed (0.041 s) among all baselines. The hybrid safety mechanism ensures a robust MOC of 3.5 ± 0.21 m, effectively preventing current-driven drift toward obstacles. Furthermore, the reward convergence curve in Figure 14b confirms that H_RS_TD3 maintains a stable and high reward level, avoiding the severe fluctuations and performance collapses observed in DDPG.

### 5.3. Dynamic Obstacle Map Experiments

**Figure 15:**
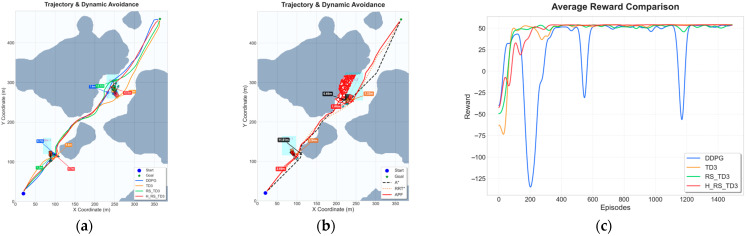
Trajectory and reward comparison on dynamic map with two vessels: (**a**) trajectory comparison of reinforcement learning–based algorithms; (**b**) trajectory comparison of traditional algorithms; (**c**) reward comparison on dynamic map with two vessels.

**Figure 16:**
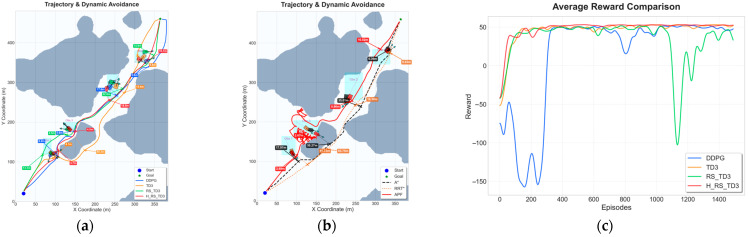
Trajectory and reward comparison on dynamic map with four vessels: (**a**) trajectory comparison of reinforcement learning–based algorithms; (**b**) trajectory comparison of traditional algorithms; (**c**) reward comparison on dynamic map with four vessels.

**Figure 17:**
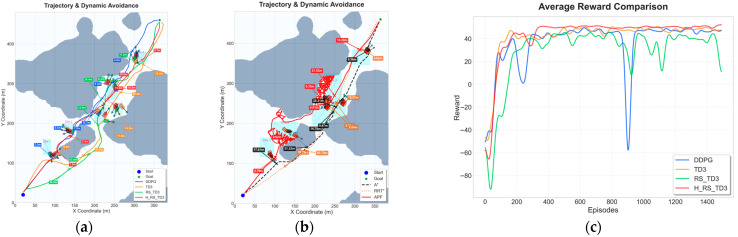
Trajectory and reward comparison on dynamic map with six vessels: (**a**) trajectory comparison of reinforcement learning–based algorithms; (**b**) trajectory comparison of traditional algorithms; (**c**) reward comparison on dynamic map with six vessels.

To assess the decision-making capability of the proposed algorithm in unstructured and highly interactive environments, a series of dynamic obstacle scenarios with increasing complexity was constructed, involving 2, 4, and 6 moving vessels, respectively. These scenarios require the USV to continuously resolve spatiotemporal conflicts while maintaining safety and progress toward the goal. The resulting trajectories and reward convergence curves are summarized in Figure 15, Figure 16 and Figure 17, with quantitative performance metrics reported in Table 8, Table 9 and Table 10.

In the two-vessel scenario shown in Figure 15, H_RS_TD3 demonstrates superior proactive behavior by generating a smooth, decisive path with the shortest length of 565.9 ± 0.4 m. While traditional planners like A* and RRT* exhibit reactive detours and APF suffers from local minima, H_RS_TD3 achieves high real-time efficiency with an inference time of 0.0349 s. According to Table 8, the algorithm maintains a high MOC of 4.5 ± 0.3 m, significantly exceeding those of the DDPG or RS_TD3 baselines. Furthermore, Figure 15c confirms that H_RS_TD3 maintains stable reward convergence throughout training, effectively avoiding the performance collapses and oscillations observed in DDPG.

As environmental congestion increases in the four-vessel scenario shown in Figure 16, behavioral differences among the planning strategies become more pronounced. As illustrated in Figure 16a,b, traditional planners and baseline reinforcement learning methods like TD3 and RS_TD3 increasingly favor conservative avoidance strategies or suffer from local minima, resulting in elongated and inefficient paths. By comparison, H_RS_TD3 effectively exploits transient spatiotemporal gaps between the moving obstacles to produce a much more direct trajectory. Quantitative results in Table 9 corroborate this advantage, showing that H_RS_TD3 achieves the shortest average path length of 574.9 ± 1.9 m while maintaining an MOC of 3.52 ± 0.37 m and a rapid inference time of 0.0417 s. Furthermore, the reward convergence curve in Figure 16c highlights that while RS_TD3 suffers from noticeable performance regression in later training stages, H_RS_TD3 maintains stable and high reward levels without degradation, demonstrating the effectiveness of prioritizing high-value interaction experiences.

In the most challenging six-vessel scenario, the advantages of proactive conflict resolution become critically apparent. As illustrated in Figure 17a,b, baseline planners exhibit excessive detouring, local minima trapping (e.g., APF), and oscillatory behavior under high interaction density, whereas H_RS_TD3 successfully navigates through the congested waterways with smooth, controlled maneuvering. Quantitative metrics in Table 10 demonstrate that H_RS_TD3 achieves the optimal balance of efficiency and safety, attaining the shortest average path length of 574.6 ± 0.86 m and avoiding the overly conservative paths generated by RS_TD3 (628.9 ± 5.0 m). Furthermore, it maintains an MOC of 2.4 ± 0.8 m while executing decisions in just 0.0458 s. Finally, the reward convergence curve in Figure 17c confirms that H_RS_TD3 effectively resists the severe instability, negative reward plunges, and late-stage performance regression seen in DDPG and RS_TD3, ensuring robust and stable navigation even in densely populated marine environments.

### 5.4. Dynamic Obstacle Experiments with Ocean Current Interference

**Figure 18:**
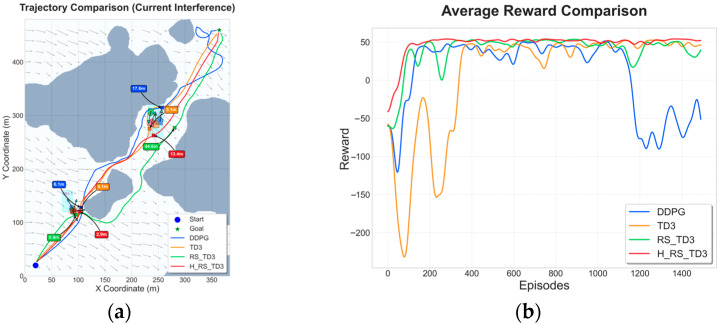
Trajectory and reward comparison on dynamic map with two vessels and ocean currents: (**a**) trajectory comparison on dynamic map with two vessels and ocean currents; (**b**) reward comparison on dynamic map with two vessels and ocean currents.

**Figure 19:**
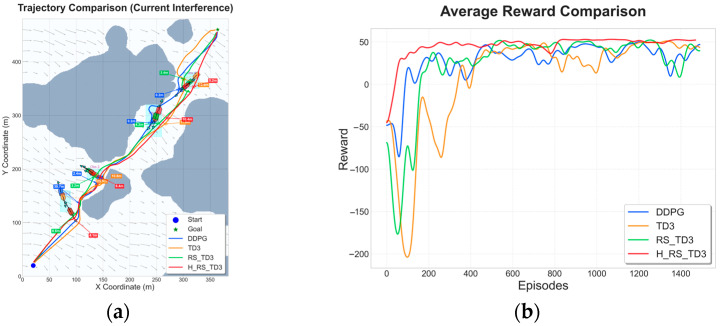
Trajectory and reward comparison on dynamic map with four vessels and ocean currents: (**a**) trajectory comparison on dynamic map with four vessels and ocean currents; (**b**) reward comparison on dynamic map with four vessels and ocean currents.

**Figure 20:**
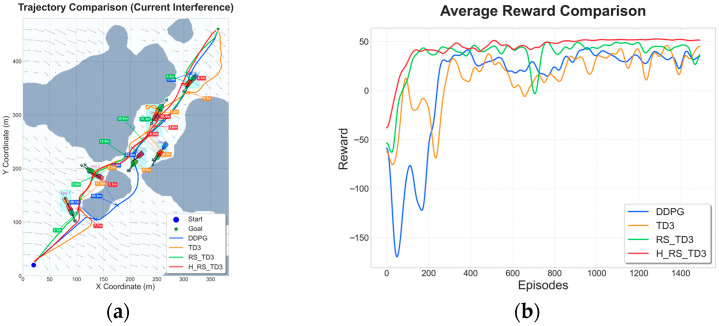
Trajectory and reward comparison on dynamic map with six vessels and ocean currents: (**a**) trajectory comparison on dynamic map with six vessels and ocean currents; (**b**) reward comparison on dynamic map with six vessels and ocean currents.

To evaluate the proposed algorithm under extreme conditions, dynamic obstacle scenarios were coupled with time-varying ocean currents derived from CMEMS data. This challenging setting requires the USV to simultaneously resolve spatiotemporal conflicts and compensate for continuous hydrodynamic drift. Because classical planners struggle with such high-dimensional coupled disturbances, this section focuses exclusively on comparing deep reinforcement learning baselines, with the overall quantitative results and visual comparisons summarized in Table 11, Table 12 and Table 13 and Figure 18, Figure 19 and Figure 20.

In the two-vessel scenario under current interference, baseline methods exhibit significant sensitivity to hydrodynamic drift. As shown in Figure 18a, DDPG frequently deviates toward obstacle boundaries, while TD3 and RS_TD3 display delayed corrective maneuvers. In contrast, H_RS_TD3 generates a smoother trajectory by proactively anticipating both obstacle motion and flow-induced displacement. Quantitative results in Table 11 confirm that H_RS_TD3 achieves the most consistent path length (568.2 ± 1.56 m) and the highest MOC (2.89 ± 0.17 m), while preserving a real-time inference speed of 0.068 s. Furthermore, the reward convergence curve in Figure 18b demonstrates that H_RS_TD3 rapidly stabilizes, effectively avoiding the severe reward oscillations experienced by DDPG and TD3 under coupled disturbances.

In the four-vessel scenario, the challenges of coupled disturbances become more pronounced. As illustrated in Figure 19a, baseline methods exhibit frequent oscillations and reactive detours, whereas H_RS_TD3 maintains a coherent trajectory through dynamically evolving gaps. Quantitative results in Table 12 corroborate this advantage: H_RS_TD3 achieves the fastest inference time (0.066 s) and a significantly larger MOC (3.92 ± 0.21 m) compared to baselines (≤2.23 m), while keeping a competitive path length (579.4 ± 1.38 m). Finally, the reward curve in Figure 19b highlights that H_RS_TD3 achieves stable convergence, successfully overcoming the severe instability and negative reward periods that plague DDPG and TD3.

In the extreme six-vessel scenario with ocean currents, baseline algorithms suffer substantial degradation and generate trajectories that dangerously approach moving obstacles, as illustrated in Figure 20a. Quantitative results in Table 13 confirm this failure; the MOC for all baselines drops critically below 1 m (e.g., 0.36 ± 0.11 m for RS_TD3). In stark contrast, H_RS_TD3 successfully preserves navigation safety with a commanding MOC of 5.20 ± 0.31 m, while concurrently achieving the fastest inference time (0.067 s) and a highly consistent path length (584.3 ± 1.22 m). Finally, the reward curve in Figure 20b demonstrates that H_RS_TD3 achieves stable and monotonic convergence, completely overcoming the severe policy instability and prolonged negative reward plunges that collapse the baseline methods under extreme coupled disturbances.

Furthermore, compared to similar dynamic obstacle avoidance experiments in the recent literature [32], which report average inference times of 30–50 ms and MOCs ranging from 1.65 m to 6.00 m, the proposed H_RS_TD3 algorithm achieves highly competitive performance across Table 8, Table 9, Table 10, Table 11, Table 12 and Table 13 (Infer Time: 34.9–68.0 ms, MOC: 2.4–5.2 m under extreme multi-vessel conditions). These findings confirm that the proposed algorithm effectively overcomes the real-time collision avoidance and hydrodynamic disturbance challenges raised in the introduction.

### 5.5. Ablation Study and Component Analysis

To verify the necessity of each component, an ablation study is conducted by comparing the full H_RS_TD3 with three variants: NonAPF (removing potential field guidance), NonTPN (removing the motion prediction network), and Normal_RB (utilizing a standard replay buffer). This configuration quantifies how individual modules contribute to reactive safety, predictive efficiency, and overall path optimization.

**Figure 21:**
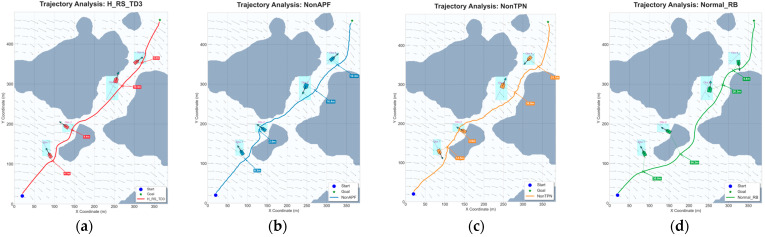
Trajectory comparison of the ablation study in dynamic underwater environments with ocean currents: (**a**) Trajectory of the proposed H_RS_TD3 algorithm; (**b**) Trajectory of the variant without the APF module; (**c**) Trajectory of the variant without the TPN module; (**d**) Trajectory of the variant using a Normal Replay Buffer instead of the prioritized/her mechanism.

Table 14 and Figure 21 show the technical trade-offs, NonAPF lacks proactive repulsion, its MOC drops to 0.87 ± 0.35 m because the vessel sails too close to land boundaries, NonTPN removes neural overhead, it reaches the fastest inference at 0.045 s, it cannot anticipate obstacle motion, Figure 21c shows reactive oscillations and a longer 590.5 ± 2.88 m path, Normal_RB results in the longest 597.3 ± 5.20 m trajectory, its high 4.42 ± 0.28 m MOC comes from an inefficient, conservative detour rather than optimized planning, H_RS_TD3 reaches the best balance, it keeps a stable 3.92 ± 0.21 m safety margin with the shortest 579.4 ± 1.38 m path.

## 6. Conclusions

This paper investigated the problem of USV path planning in complex and time-varying marine environments and proposed an improved deep reinforcement learning framework, termed H_RS_TD3, which integrates hybrid potential field constraints with trajectory prediction. The navigation task was formulated as an MDP. To address the limitations of conventional and learning-based approaches in unstructured environments, the proposed framework incorporates key components, including CDA-PER, TPN, and Current-Aware Compensation, to enhance learning efficiency, safety, and robustness in unstructured marine environments.

Comprehensive simulations were conducted across a wide range of scenarios, including static island-rich maps, dynamic multi-vessel encounters, and coupled environments with non-uniform, time-varying ocean currents derived from CMEMS data. The experimental results demonstrate that H_RS_TD3 consistently outperforms classical planners (A*, RRT*, and APF) and mainstream deep reinforcement learning baselines (DDPG and TD3) in terms of path efficiency, safety, and stability.

The main advantages of the proposed method can be summarized as follows:

1. Enhanced learning efficiency is achieved through the CDA-PER mechanism, which dynamically prioritizes experiences based on both temporal-difference error and trajectory curvature characteristics. This design significantly accelerates policy convergence in sparse-reward environments and mitigates late-stage performance degradation commonly observed in complex interactive scenarios. Across all dynamic tests, H_RS_TD3 exhibits the lowest path-length variance, indicating stable and consistent planning behavior.
2. Superior safety performance is realized by combining trajectory prediction with hybrid potential field constraints. This integration enables a transition from purely reactive avoidance to proactive conflict resolution. Even in the most challenging scenarios involving six moving vessels and strong ocean currents, H_RS_TD3 maintains a substantially larger minimum obstacle clearance compared to baseline methods, which frequently approach unsafe proximity under coupled disturbances.
3. The proposed framework preserves real-time feasibility. Despite the inclusion of auxiliary prediction modules, the inference latency of H_RS_TD3 remains within 17.3 ms to 68.0 ms per decision step across all scenarios (as detailed in Table 4, Table 5, Table 6, Table 7, Table 8, Table 9, Table 10, Table 11, Table 12 and Table 13). This computational efficiency, achieved on a workstation equipped with an Intel i7-12700F CPU and an RTX 3060 Ti GPU, corresponds to an operational frequency of approximately 15–50 Hz. The reduction in decision uncertainty provided by trajectory prediction effectively suppresses oscillatory control behavior, allowing the algorithm to meet the real-time requirements of high-speed USV navigation.
4. H_RS_TD3 demonstrates robust performance across increasing environmental complexity. The algorithm generalizes well from static environments to highly dynamic and disturbance-rich conditions, successfully compensating for lateral drift induced by non-uniform ocean currents without suffering from local minima or excessive path redundancy, which commonly affect potential-field-based methods. These results highlight the practical applicability of the proposed framework in realistic marine environments.

In summary, the proposed H_RS_TD3 algorithm achieves a favorable balance between path efficiency, safety, and computational efficiency. While maintaining path lengths comparable to globally optimal planners in static settings, it significantly improves robustness and safety under multi-vessel interactions and strong current disturbances, addressing key challenges faced by USV path planning in real-world conditions.

Nevertheless, the present study is subject to several limitations. The simulations assume idealized global perception and perfectly observable local currents, and do not explicitly account for sensor noise or flow estimation errors. Introducing such noise would increase observation variance, potentially elongating path lengths and increasing inference times. However, the proposed uncertainty-aware mechanism and the closed-loop nature of the H_RS_TD3 algorithm are inherently designed to mitigate unpredictable hydrodynamic drift, prioritizing safe obstacle clearance even under degraded perception or environmental uncertainties.

Future work will focus on quantifying the impacts of varied signal-to-noise ratios, transferring the proposed framework from simulation to real-world USV platforms, incorporating domain randomization to enhance robustness against environmental uncertainty, and extending the approach to multi-USV systems. In particular, decentralized cooperative navigation under communication constraints will be investigated to further improve the applicability of the proposed method to large-scale maritime operations.

## Figures and Tables

**Table 1:** Parameters of dataset and simulation environment.

Category	Parameters	Symbol	Value
Dataset and Map	Static map geographic extent	/	120.76° E–120.97° E, 38.26° N–38.42° N
	Grid smoothing interpolation	/	Bilinear
	Ocean current mapping	$V_{c}$ $,\;{\hat{V}}_{c}$	Spatiotemporal grids
	Dataset date of ocean currents	/	25 November 2025
USV Physical Model	USV mass	m	100 kg
	USV length	L	2.38 m
	Maximum thrust limit	$F_{max}$	300 N
	Maximum rudder angle	$\delta_{max}$	$25^{{}^{\circ}}$
	Water density	$\rho_{w}$	1025 kg/m^3^
Perception and Control	Number of LiDAR beams	$N_{beams}$	60
	Maximum radar range	$d_{max}$	30 m
	Simulation time step	Δt	0.1 s
	Control frame skip	$k_{skip}$	4
	Current sensing mode	/	Local observed

**Table 2:** Hyperparameters of the proposed algorithm training.

Hyperparameters	Symbol	Value
Reward Discount Rate	γ	0.99
Actor Network Learning Rate	$lr_{Actor}$	$3\times 10^{-4}$
Critic Network Learning Rate	$lr_{Critic}$	$3\times 10^{-4}$
TPN Learning Rate	$lr_{TPN}$	$3\times 10^{-4}$
Soft Update Rate	τ	0.005
Experience replay storage pool size	$M_{buffer}$	100,000
Batch size for experience replay learning	N	256
Target Actor Network Update Frequency	$d_{freq}$	2
Maximum number of training sessions	M	1500
Maximum steps per episode	T	1500
Action Exploration Noise	$\sigma_{expl}$	0.2
Target Policy Noise Clip	c	0.5
Prediction Horizon	H	30
Uncertainty Penalty Coefficient	$\lambda_{u}$	0.10
CDA-PER Priority Exponent	κ	0.6
CDA-PER Importance Sampling Weight	β	0.4→1.0
CDA-PER Reward Decay Rate	$\gamma_{r}$	0.99
APF Attractive Gain	$\eta_{att}$	1.0
APF Repulsive Gain	$\eta_{static}$	0.8
APF Static Safety Radius	$d_{0}$	2.0

**Table 3:** Hardware specifications for simulation.

Component	Specification
CPU	12th Gen Intel(R) Core (TM) i7-12700F
RAM	32.0 GB (5200 MT/s)
GPU	NVIDIA GeForce RTX 3060 Ti
OS	Windows 11 64-bit

**Table 4:** Performance comparison on static Map 1.

Algorithm	A*	RRT*	APF	DDPG	TD3	RS_TD3	H_RS_TD3
Path Length (m)	566.59	562.42	558.94	572.56 ± 5.64	567.46 ± 1.94	565.82 ± 1.98	564.17 ± 0.69
Infer Time (s)	10.71	10.25	0.0424	0.0263	0.0223	0.0202	0.0173
MOC (m)	0.86	1.03	0.27	3.92 ± 0.86	6.22 ± 0.43	4.00 ± 0.56	5.77 ± 0.47

**Table 5:** Performance comparison on static Map 2.

Algorithm	A*	RRT*	APF	DDPG	TD3	RS_TD3	H_RS_TD3
Path Length (m)	475.80	464.69	479.40	473.36 ± 1.64	469.82 ± 1.33	466.00 ± 0.74	467.52 ± 0.71
Infer Time (s)	8.79	20.97	0.0846	0.0214	0.0209	0.0178	0.0180
MOC (m)	0.66	0.71	0.39	3.00 ± 0.79	2.93 ± 0.47	4.10 ± 0.66	4.14 ± 0.36

**Table 6:** Performance comparison on static Map 1 with ocean currents.

Algorithm	DDPG	TD3	RS_TD3	H_RS_TD3
Path Length/m	567.9 ± 7.34	586.7 ± 4.36	578.3 ± 5.86	566.7 ± 3.40
Infer Time/s	0.068	0.054	0.062	0.057
MOC/m	2.83 ± 0.16	2.00 ± 0.16	4.47 ± 0.16	5.10 ± 0.16

**Table 7:** Performance comparison on static Map 2 with ocean currents.

Algorithm	DDPG	TD3	RS_TD3	H_RS_TD3
Path Length/m	489.9 ± 7.96	477.5 ± 2.14	468.4 ± 2.22	468.3 ± 1.32
Infer Time/s	0.077	0.073	0.086	0.041
MOC/m	5.1 ± 0.21	2.3 ± 0.25	2.7 ± 0.18	3.5 ± 0.21

**Table 8:** Performance comparison on dynamic map with two vessels.

Algorithm	A*	RRT*	APF	DDPG	TD3	RS_TD3	H_RS_TD3
Path Length (m)	575.96	565.74	758.99	572.1 ± 0.6	579.9 ± 4.0	575.6 ± 2.6	565.9 ± 0.4
Infer Time (s)	14.99	8.1002	5.0142	0.0636	0.0393	0.0494	0.0349
MOC (m)	0.86	0.96	0.38	0.51 ± 0.8	3.88 ± 1.2	1.83 ± 0.7	4.5 ± 0.3

**Table 9:** Performance comparison on dynamic map with four vessels.

Algorithm	A*	RRT*	APF	DDPG	TD3	RS_TD3	H_RS_TD3
Path Length (m)	594.02	578.39	731.09	585.5 ± 2.2	599.6 ± 6.4	588.3 ± 3.2	574.9 ± 1.9
Infer Time (s)	14.0810	8.5169	4.43	0.0493	0.0319	0.052	0.0417
MOC (m)	0.62	0.61	0.45	3.19 ± 1.12	4.2 ± 0.17	4.38 ± 0.69	3.52 ± 0.37

**Table 10:** Performance comparison on dynamic map with six vessels.

Algorithm	A*	RRT*	APF	DDPG	TD3	RS_TD3	H_RS_TD3
Path Length (m)	598.76	579.23	993.62	579 ± 2.1	604 ± 10.4	628.9 ± 5.0	574.6 ± 0.86
Infer Time (s)	12.1001	8.22	9.9760	0.0499	0.0497	0.0354	0.0458
MOC (m)	0.86	1.17	0.34	1.1 ± 0.9	5.5 ± 0.8	6.9 ± 1.3	2.4 ± 0.8

**Table 11:** Performance comparison in dynamic map with two vessels and ocean currents.

Algorithm	DDPG	TD3	RS_TD3	H_RS_TD3
Path Length/m	609.0 ± 8.72	577.7 ± 2.92	590.9 ± 10.86	568.2 ± 1.56
Infer Time/s	0.085	0.065	0.079	0.068
MOC/m	0.49 ± 0.26	2.82 ± 0.25	2.25 ± 0.22	2.89 ± 0.17

**Table 12:** Performance comparison in dynamic map with four vessels and ocean currents.

Algorithm	DDPG	TD3	RS_TD3	H_RS_TD3
Path Length/m	593.7 ± 6.44	599.9 ± 3.86	577.0 ± 3.84	579.4 ± 1.38
Infer Time/s	0.103	0.073	0.076	0.066
MOC/m	1.50 ± 0.23	2.05 ± 0.23	2.23 ± 0.23	3.92 ± 0.21

**Table 13:** Performance comparison in dynamic map with six vessels and ocean currents.

Algorithm	DDPG	TD3	RS_TD3	H_RS_TD3
Path Length/m	595.9 ± 8.00	598.1 ± 4.30	577.6 ± 3.46	584.3 ± 1.22
Infer Time/s	0.083	0.092	0.068	0.067
MOC/m	0.71 ± 0.46	0.56 ± 0.14	0.36 ± 0.11	5.20 ± 0.31

**Table 14:** Performance comparison of ablation variants in dynamic map with ocean currents.

Algorithm	H_RS_TD3	NonAPF	NonTPN	Normal_RB
Path Length/m	579.4 ± 1.38	586.2 ± 3.15	590.5 ± 2.88	597.3 ± 5.20
Infer Time/s	0.066	0.059	0.045	0.065
MOC/m	3.92 ± 0.21	0.87 ± 0.35	2.95 ± 0.42	4.42 ± 0.28

## Data Availability

The data contained within the article.

## Abbreviations

The following abbreviations are used in this manuscript:

USV	Unmanned Surface Vehicle
H_RS_TD3	Hybrid Safety and Reward-Sensitive Twin Delayed Deep Deterministic Policy Gradient
TD3	Twin Delayed Deep Deterministic Policy Gradient
MDP	Markov Decision Process
TPN	Trajectory Predictor Network
CDA-PER	Curvature-driven Advantage-based Prioritized Experience Replay
DRL	Deep Reinforcement Learning
APF	Artificial Potential Field
DDPG	Deep Deterministic Policy Gradient
